# Aberrant activation of Notch signaling in extrahepatic cholangiocarcinoma: clinicopathological features and therapeutic potential for cancer stem cell-like properties

**DOI:** 10.1186/s12885-016-2919-4

**Published:** 2016-11-07

**Authors:** Shuichi Aoki, Masamichi Mizuma, Yayoi Takahashi, Yoichi Haji, Ryo Okada, Tomoya Abe, Hideaki Karasawa, Keiichi Tamai, Takaho Okada, Takanori Morikawa, Hiroki Hayashi, Kei Nakagawa, Fuyuhiko Motoi, Takeshi Naitoh, Yu Katayose, Michiaki Unno

**Affiliations:** 1 Department of Surgery, Tohoku University Graduate School of Medicine, 1-1 Seiryomachi, Aobaku, Sendai, 980-8574 Japan; 2 Department of Pathology, Tohoku University Hospital, 1-1 Seiryomachi, Aobaku, Sendai, 980-8574 Japan; 3 Division of Cancer Stem Cell, Miyagi Cancer Center Research Institute, 47-1 Nodayama, Medeshimashiote aza, Natori, 981-1293 Japan

**Keywords:** Notch signaling, Cholangiocarcinoma, Hes-1, Cancer stem cell

## Abstract

**Background:**

Little is known about the roles of Notch signaling in cholangiocarcinoma (CC). The expression of hairy and enhancer of split 1 (Hes-1) has not been investigated yet in resected specimens of CC. Notch signaling has been reported to be related to cancer stem cell (CSC) like properties in some malignancies. Our aim is to investigate the participation of Notch signaling in resected specimens of extrahepatic CC (EHCC) and to evaluate the efficacy of CC cells with CSC-like properties by Notch signaling blockade.

**Methods:**

First, the expression of Notch1, 2, 3, 4 and Hes-1 was examined by immunohistochemistry in 132 resected EHCC specimens. The clinicopathological characteristics in the expression of Notch receptors and Hes-1 were investigated. Second, GSI IX, which is a γ-secretase-inhibitor, was used for Notch signaling blockade in the following experiment. Alterations of the subpopulation of CD24^+^CD44^+^ cells, which are surface markers of CSCs in EHCC, after exposure with GSI IX, gemcitabine (GEM), and the combination of GSI IX plus GEM were assessed by flow cytometry using the human CC cell lines, RBE, HuCCT1 and TFK-1. Also, anchorage-independent growth and mice tumorigenicity in the cells recovered by regular culture media after GSI IX exposure were assessed.

**Results:**

Notch1, 2, 3, 4 and Hes-1 in the resected EHCC specimens were expressed in 50.0, 56.1, 42.4, 6.1, and 81.8 % of the total cohort, respectively. Notch1 and 3 expressions were associated with poorer histological differentiation (*P* = 0.008 and 0.053). The patients with the expression of at least any one of Notch1-3 receptors, who were in 80.3 % of the total, exhibited poorer survival (*P* = 0.050). Similarly, the expression of Hes-1 tended to show poor survival (*P* = 0.093). In all of the examined CC cell lines, GSI IX treatment significantly diminished the subpopulation of CD24^+^CD44^+^ cells. Although GEM monotherapy relatively increased the subpopulation of CD24^+^CD44^+^ cells in all lines, GSI IX plus GEM attenuated it. Anchorage-independent growth and mice tumorigenicity were inhibited in GSI IX-pretreated cells in RBE and TFK-1 (*P* < 0.05).

**Conclusion:**

Aberrant Notch signaling is involved with EHCC. Inhibition of Notch signaling is a novel therapeutic strategy for targeting cells with CSC-like properties.

## Background

Cholangiocarcinoma (CC) arises from epithelial cells lining the bile duct. The incidence of CC is the highest in East and South Asia and has been increasing worldwide [1, 2]. Chronic damage and inflammation of the biliary epithelium, such as from gallstones, chronic hepatitis, primary sclerosing cholangitis and liver fluke infection, are considered risk factors for the formation of CC [3, 4]. Although various genetic alterations in CC have been reported [5–7], molecular biological information about CC is scant. Complete surgical resection offers the only chance for cure [8]. Nevertheless, the prognosis after surgery for CC is poor, especially for advanced tumors, such as node metastasis and perineural invasion. The efficacy of chemotherapy for CC, in which the combination therapy of gemcitabine (GEM) and cisplatin (CDDP) is now considered the best for advanced CC [9, 10], is limited in its ability to cure malignancies. Therefore, the emergence of a novel therapeutic strategy is urgently needed.

Notch signaling is an evolutionarily conserved pathway that plays an important role in various cellular and developmental processes [11]. Aberrant activation of Notch signaling has been shown to be involved in various malignancies, such as pancreatic cancer, breast cancer, lung cancer and leukemia [12–15]. Concerning CC, which is classified into intrahepatic CC (IHCC) or extrahepatic CC (EHCC) according to the primary site, only a few reports have demonstrated the aberrant expression of Notch receptors or ligands by immunohistochemistry (IHC) using clinically resected specimens of EHCC or IHCC [16, 17]. In IHCC, the aberrant expressions of Notch1 and Notch4 were reported to be associated with cancer progression [16]. On the other hand, the aberrant expression of Notch1 and 3 correlated with cancer progression in EHCC [17]. However, in both reports, the presence of aberrant hairy and enhancer of split 1 (Hes-1) expression, which is a representative downstream target gene of Notch signaling, had not been evaluated. According to recent studies using transgenic mice, consecutive Notch1 or 2 signaling induced the formation of IHCC by the liver progenitor cells [18–20]. Thus, further studies are needed to elucidate a role of Notch signaling, including types of Notch receptors, for CC.

Notch signaling is initiated by ligand binding from adjacent cells, followed by intramembranous proteolytic cleavage of the Notch receptor by the γ-secretase complex and release of the Notch intracellular domain (NICD). NICD translocates to the nucleus and induces target genes, such as *Hes-1*. γ-secretase inhibitor (GSI) has been reported to have antitumor effects as Notch antagonism by suppression of the Notch receptor cleavage against cancers linked with aberrant activation of Notch signaling in vitro and in vivo [13, 14, 21, 22]. Clinical trials for GSI for some malignancies are ongoing [23, 24]. Cancer stem cells (CSCs), which are critical for tumor initiation, progression and persistence, are considered to be generally resistant to conventional chemotherapy. Notch signaling plays a pivotal role in the initiation and maintenance of tumor [25–27]. Although several reports described that blocking Notch signaling by GSI showed inhibition of cell proliferation and invasion in CC in vitro [28, 29], the efficacy of GSI for CC cells with CSC-like properties has not been confirmed.

The aim of the present study is two-fold. First, we investigated the correlation between the expression of Notch1, 2, 3, 4 or Hes-1 and clinicopathological factors using resected EHCC specimens. Second, therapeutic effectiveness of GSI for cells with CSC-like properties was evaluated using CC cell lines.

## Methods

### Patient selection and specimens

One hundred thirty-two consecutive patients with surgically resected EHCC at our institution between 2000 and 2008, who did not receive chemotherapy or radiotherapy before surgery, were examined in this study. The medical records including clinicopathological findings and paraffin-embedded tissues of resected EHCC were collected for all patients. Pathological diagnosis was done by two pathologists with expertise in hepato-biliary-pancreatic pathology. Histological differentiation and tumor staging were based on the 7th edition of Union for the International Cancer Control (UICC) classification. When local recurrence or distant metastasis was present, chemotherapies and/or radiation therapies were applied to patients with good performance status 0–2 (Eastern Cooperative Oncology Group, ECOG). This study was approved by the Institutional Review Board of Tohoku University. We obtained written informed consent for participation in the study from all of the patients.

Most patients with EHCC received biliary drainage due to biliary obstruction during the preoperative period. Biliary drainage generally causes inflammatory changes of non-neoplastic cholangiocytes, which often induces the expression of Notch receptors in it [17, 30]. Therefore, normal bile duct tissues of 8 patients with pancreatic neuroendocrine tumors (pNET) who underwent pancreaticoduodenectomy were assessed as controls. Survival analysis was performed in patients with R0 resection.

### Immunohistochemistry (IHC)

IHC was performed using antibodies of Notch1 (sc-6014, dilution 1:100, Santa Cruz Biotechnology, Inc., TX, USA), Notch2 (sc-5545, dilution 1:200, Santa Cruz Biotechnology, Inc.), Notch3 (sc-5593, dilution 1:500, Santa Cruz Biotechnology, Inc.), and Notch4 (sc-5594, dilution 1:200, Santa Cruz Biotechnology, Inc.) receptors and Hes-1 (ab49170, dilution 1:200, Abcam plc, Cambridge, UK). Concerning the immunostaining method by antibodies of Notch1, 2, 3 and 4, the streptavidin-biotin (SAB) method was applied. Briefly, sections of 2 μm thick from a paraffin-embedded tissue blocks were deparaffinized in xylene for 10 min, rehydrated using a graded alcohol series, placed in an endogenous peroxide blocker for 10 min and washed with buffer. The slides were microwaved for 15 min (Notch1), autoclaved for 5 min (Notch2 and 3) or trypsinized for 30 min (Notch4) for antigen retrieval. Primary antibodies of Notch1, 2, 3 and 4 were applied overnight at 4 °C and antibody binding was detected using biotinylated anti-goat or anti-rabbit IgG conjugating streptavidin-peroxidase complex (BA-9500, Vector Laboratories, CA, USA) (Histofine SAB-PO® kit, Nichirei Bioscience Inc., Tokyo, Japan) for 30 min. Finally, the sections were developed with 3,3′-diaminobenzidine color solution for 3 min at room temperature. Then, hematoxylin was used as a chromogen and the slides were consecutively counter-stained for 60 s.

### Interpretation for IHC

**Fig. 1:**
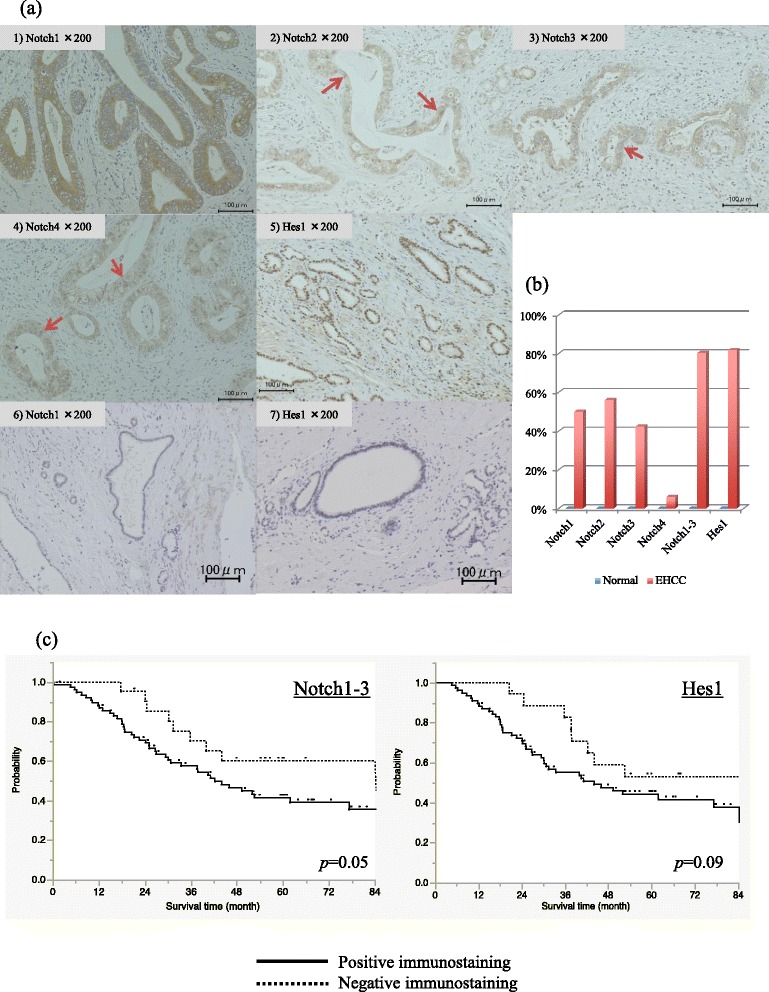
IHC of Notch receptors and Hes-1 in resected EHCC specimen. **a** Representative photographs of IHC. 1), 2), 3), 4) and 5) are Notch1, 2, 3, 4 and Hes-1, respectively, in the EHCC. 6) and 7) are Notch1 and Hes-1, respectively, in the normal bile duct of resected pNET specimens. Although Notch1, 2, 3, 4 and Hes-1 were stained very weakly in the non-neoplastic biliary cytoplasm or nuclear (**a**-5,6), the cytoplasmonuclear coexistent localization of Notch receptors (*arrow*) was defined as positive staining (**a**-1-4). The cases with nuclear expression of Hes-1 in more than 70 % of the tumor cells per tumor were defined as positive (**a**-5). **b** Expression rate of Notch receptors and Hes-1 in EHCC and normal bile ducts. Of 132 resected EHCC specimens, there was positive immunostaining of Notch1, 2, 3, 4 and Hes-1 in 66 (50.0 %), 74 (56.1 %), 56 (42.4 %), 8 (6.1 %) and 108 (81.8 %) specimens, respectively, and cases with positive immunostaining in at least any one of Notch1, 2 and 3 were shown in 106 specimens (80.3 %). **c** Overall survival curve of EHCC patients with R0 resection. Patients with at least one expression of any Notch1-3 exhibited poorer prognosis than those with no expression of Notch1, 2 or 3 (3-years OS: 57.6 % vs 70.2 %, *P* = 0.050). Hes-1 expression tended to be related to poorer prognosis (3-years OS: 55.1 % vs 82.6 %, *P* = 0.093)

Although Notch1, 2, 3 and 4 were stained very weakly in the non-neoplastic biliary cytoplasm of some cases, the cytoplasmonuclear coexistent localization of Notch receptors (Fig. 1a: arrow) was defined as positive staining, as like the previous report [17]. Only either the cytoplasmic or nuclear stained cases were defined as negative. The cases with nuclear expression of Hes-1 in more than 70 % of the tumor cells per tumor were defined as positive. Witten informed consent for the publication of Fig. 1a was obtained from all of the patients.

### Cell culture

Human CC cell lines (RBE, HuCCT1 and TFK-1) were used for this study. RBE was obtained from RIKEN Bio Resource Center (Tsukuba, Japan). HuCCT1 and TFK-1 were obtained from the Cell Resource Center for Biomedical Research of Tohoku University. All cell lines were cultured in RPMI 1640 medium (Sigma Aldrich, MO, USA) supplemented with 10 % heat-inactivated fetal bovine serum (FBS) (Sigma Aldrich) and 1 % penicillin/streptomycin (Thermo Fisher Scientific Inc., MA, USA) at 37 °C in 5 % CO_2_.

### Drugs and treatment in vitro

GSI IX (Merck Millipore, MA, USA) was prepared as a 10 mM stock solution in dimethyl sulfoxide (DMSO) (Wako Pure Chemical Industries, Ltd., Osaka, Japan) and was diluted with media before the treatment in vitro. Cells were treated with GSI IX (20 or 40 μM) or DMSO as a control and then analyzed. GEM (LKT Laboratories, Inc., MN, USA) was used at 40nM in solution with phosphate buffered saline (PBS).

### Quantitative real-time reverse transcription polymerase chain reaction (qPCR)

Cells cultured with GSI IX or DMSO for 72 h were then evaluated. Total RNA was isolated using NucleoSpin RNA II (Takara Bio Inc., Shiga, Japan) and analyzed by nanodrop (Thermo Fisher Scientific Inc.). qPCR was carried out using StepOnePlus real-time PCR system (Thermo Fisher Scientific Inc.) using SYBR Premix Ex Taq II (Tli RNaseH Plus) (Takara Bio Inc.). *GAPDH* was used as a housekeeping gene. qPCR was done at the annealing temperature of 60 °C with the following primers for *GAPDH*: 5′-GCACCGTCAAGGCTGAGAAC-3′ for sense and 5′-TGGTGAAGACGCCAGTGGA-3′ for antisense and for *Hes-1*: 5′-TCAGCTGGCTCAGACTTTCA-3′ for sense and 5′-TCAACACGACACCGGATAAA-3′ for antisense. Relative amount of mRNA was calculated by the 2^−ΔΔCT^ method.

### Protein extraction and Western blotting

Cells cultured with GSI IX or vehicle for 96 h were lysed in lysis buffer containing 1 mM Phenylmethanesulfonyl Fluioride (PMSF) (Cell signaling technology Inc., MA, USA). For immunoblotting, the cell lysates were loaded on a 4 to 15 % sodium dodecyl sulfate (SDS)-polyacrylamide gel at equal amounts of protein (20 μg) per well and transferred to Polyvinylidenefluoride (PVDF) membranes using Trans-Blot Turbo Blotting System (Bio-Rad, CA, USA). The membranes were blocked using SuperBlock (TBS) Blocking Buffer (Thermo Fisher Scientific Inc.) for 1 h at room temperature. Then, they were probed with primary antibodies against cleaved Notch 1 (#4147, dilution 1:1000, Cell signaling technology Inc.), Hes-1 (#11988, dilution 1:1000, Cell signaling technology Inc.) and GAPDH (#5174, dilution 1:1000; Cell signaling technology Inc.). The signals were detected by Clarity Western ECL Substrate (Bio-Rad) according to the manufacturer’s instructions.

### Proliferation assay

In order to investigate the effect of GSI IX on cell proliferation, cells were plated at a concentration of 1 × 10^3^ cells/well in a 96 well plate overnight. Afterward, cells were treated with DMSO, different concentrations of GSI IX (20 and 40 μM) and combination of GSI IX (40 μM) and GEM (40nM), and measured at different time points (1–4 days). At the respective time point, 10 μL water-soluble tetrazodium salt (Cell Counting Kit-8 Reagent) (DOJINDO LABORATORIES, Kumamoto, Japan) was added to each well and incubated for 2 h at 37 °C. The absorbance was detected at a wavelength of 490 nm.

### Flow cytometric analyses

Flow cytometric analysis was performed using a FACSAria II (Becton Dickinson Biosciences, CA, USA), with antibodies CD24-BV421 and CD44-APC (BD Biosciences), previously described [31]. In brief, dissociated cells were counted at a concentration of 10^6^ cells per 100 μL in a 5 ml tube, washed and resuspended in PBS buffer containing 0.5 % bovine serum albumin (BSA) and 2 mM ethylenedinitrilotetraacetic acid (EDTA). Cells with higher-expressing levels of CD24 or CD44 than those seen in IgG controls (BD Biosciences) were considered positive. Side scatter and forward scatter profiles were used to eliminate cell doublets. Cells were exposed with DMSO, GSI, GEM or GSI plus GEM for 96 h. Experiments were repeated three times for each line.

### Anchorage-independent growth

The anchorage-independent growth of cells was investigated using soft agar assays. Briefly, cells were incubated in media containing 0.5 % FBS with DMSO or GSI IX (20 or 40 μM) for 96 h. Afterward, the treated cells were recovered from the media with 10 % FBS for 24 h. Then, 1 × 10^4^ viable cells from each condition were seeded in 6-well plates for soft agar assays. Viable cells were quantified using a hemocytometer with trypan blue counterstain. A bottom layer of 1 % agarose (Thermo Fisher Scientific Inc.), a middle layer of 0.6 % agarose and a top layer of medium alone were applied in each well. After incubating the plates for 8 weeks, colonies were stained with crystal violet solution and quantified by counting the number of colonies in 9 random fields at 5× magnification.

### Engraftment of ex vivo pretreated CC cells in immunodeficiency mice

All animal experiments conformed to the guidelines of the Institutional Animal Care and Use Committee of Tohoku University and were performed in accordance with the Guide for the Care and Use of Laboratory Animals of Tohoku University.

Nonobese diabetic/severe combined immunodeficiency (NOD/SCID) female mice were purchased from CLEA Japan, Inc. (Tokyo, Japan). CC cell lines were pretreated ex vivo with media containing 0.5 % FBS with DMSO or GSI IX (40 μM) for 96 h, followed by recovery in full serum conditions for an additional 24 h before subcutaneous implantation. Viable 3 × 10^6^ cells in a total volume of 200 μL of 1:1 (v/v) PBS/Matrigel (BD Biosciences) were subcutaneously inoculated into bilateral flanks (right flank: DMSO-pretreated cells, left flank: GSI IX-pretreated cells) of mice (*N* = 6). These tumors were measured every 10 days using an electronic caliper (A&D Company Ltd., Tokyo, Japan). The tumor volume was calculated using the following formula [31]: Tumor Size = [Length × Width^2^]/2.

### Statistical analysis

The *χ* ^2^ test was used to compare categorical variables and the Kaplan-Meier method was used to generate survival curves. The association between clinicopathological factors and Notch receptors/Hes-1 was assessed with the Pearson correlation coefficient. Analyzed data were described as the mean ± S.E. A Wilcoxon test was used for statistical analysis with JMP Pro 11.2.0 (SAS Institute Inc., NC, USA). Significant difference between experimental groups was determined as a *P*-value < 0.05.

## Results

### Clinicopathological characteristics of patients

One hundred thirty-two patients with EHCC, comprising 92 men and 40 women (median age: 68 y.o.), were diagnosed with 83 perihilar and 49 distal CC. Among the 132 patients, pathological arterial and portal invasion was observed in 6 and 22 patients, respectively (Table 1). According to the histological differentiation, the number of patients with grade 2 (*n* = 100: 75.8 %) was the highest. Lymph node metastases were observed in 91 patients (68.9 %). The number of patients with Stage I, II, III and IV were 20 (15.2 %), 57 (43.2 %), 18 (13.6 %) and 37 (28.0 %), respectively. R0 resection was achieved in 98 patients (74.2 %).

**Table 1:** Clinicopathological factors of EHCC patients

		Number	Percent
Total		132	100.0 %
Age	Median	68	
	Range	15–83	
Gender	Male	92	69.7 %
	Female	40	30.3 %
Location	Perihilar	83	62.9 %
	Distal	49	37.1 %
Arterial invasion	Yes	6	4.5 %
	No	126	95.5 %
Portal invasion	Yes	22	16.7 %
	No	110	83.3 %
Histopathological grading	1	20	15.2 %
	2	100	75.8 %
	3	12	9.1 %
Tumor	1	4	3.0 %
	2	63	47.7 %
	3	34	25.8 %
	4	31	23.5 %
Node	1	91	68.9 %
	0	41	31.1 %
Stage	I	20	15.2 %
	II	57	43.2 %
	III	18	13.6 %
	IV	37	28.0 %
Residual tumor classification	0	98	74.2 %
	1 or 2	34	25.8 %

### Expression of Notch receptors and Hes-1 in the resected specimens

Of 132 resected EHCC specimens, there was positive immunostaining of Notch1, 2, 3, 4 and Hes-1 in 66 (50.0 %), 74 (56.1 %), 56 (42.4 %), 8 (6.1 %) and 108 (81.8 %) specimens, respectively, and cases with positive immunostaining in at least any one of Notch1, 2 and 3 were shown in 106 specimens (80.3 %) (Fig. 1b). On the other hand, in normal cholangiocytes of resected pNET specimens, no positive immunostaining of Notch1, 2, 3, 4 and Hes-1 was observed (Fig. 1a 6)7), b).

### Clinicopathological factors and prognosis in expressions of Notch receptors and Hes-1

The number of patients with Notch1 expression was significantly greater in those with Grade 2/3 than Grade 1 (*P* = 0.008) (Table 2). Cases with the expression of Notch3 were also significantly more common in Grade 2/3 than in Grade 1 (*P* = 0.053). In terms of the Tumor category, T1/2 was higher than T3/4 in the expression of Noch3 (*P* = 0.049). According to the stage classification, there was no significant difference in the expression of any Notch receptors and Hes-1. 92 specimens of cases with at least one expression of Notch1, 2 and 3 (86.8 %) also showed positive staining of Hes1 and there was significant correlation between them (*P* = 0.005). By Pearson’s correlation analysis, there was no significant correlation between the clinicopathological factors and expression of Notch receptors/Hes-1.

**Table 2:** Expression of Notch receptors and Hes-1 in EHCC patients

		Notch1				Notch2			
		+	−	*p*	*r* ^*2*^	+	−	*p*	*r* ^*2*^
Total (%)		66 (50.0)	66 (50.0)			74 (56.1)	58 (43.9)		
Histopathological grading	G1	5	16	0.008	0.186	10	11	0.397	0.019
	G2/3	61	50			64	47		
Tumor	1/2	35	32	0.259	0.089	36	31	0.584	0.067
	3/4	31	34			38	27		
Node	0	46	46	1.000	0.000	49	43	0.324	0.066
	1	20	20			25	15		
Stage	I/II	41	36	0.377	−0.086	39	38	0.776	0.123
	III/IV	25	30			35	20		
		Notch3				Notch4			
		+	−	*p*	*r* ^*2*^	+	−	*p*	*r* ^*2*^
Total (%)		56 (42.4)	76 (57.6)			8 (6.1)	124 (93.9)		
Histopathological grading	G1	5	16	0.053	0.186	0	21	0.090	0.161
	G2/3	52	59			8	103		
Tumor	1/2	34	33	0.049	−0.134	5	62	0.491	0.007
	3/4	22	43			3	62		
Node	0	36	56	0.247	0.091	5	87	0.654	0.040
	1	20	20			3	37		
Stage	I/II	33	44	0.929	0.013	4	73	0.624	0.049
	III/IV	24	31			4	51		
		Notch1-3				Hes1			
		+	−	*p*	*r* ^*2*^	+	−	*p*	*r* ^*2*^
Total (%)		106 (80.3)	26 (19.7)			108 (81.8)	24 (18.2)		
Histopathological grading	G1	15	6	0.283	0.056	16	4	0.951	0.022
	G2/3	91	20			92	20		
Tumor	1/2	52	15	0.430	0.043	54	13	0.712	0.079
	3/4	54	11			54	11		
Node	0	71	21	0.156	0.119	73	19	0.371	0.097
	1	35	5			35	5		
Stage	I/II	59	18	0.202	0.130	60	17	0.163	0.058
	III/IV	47	8			48	7		

*P* value: *χ* ^2^ test

*r* ^2^ value: Pearson correlation coefficient

In the 98 patients with R0 resection, there was no significant survival difference between patients with and without the expression of each Notch receptor (data not shown). However, those with at least one expression of Notch1, 2 and 3 exhibited a poorer prognosis than those with no expression of Notch1, 2 or 3 (3-years overall survival (OS): 57.6 % vs 70.2 %, *P* = 0.050) (Fig. 1c). Similarly, patients with Hes-1 expression tended to show a worse prognosis than those without Hes-1 expression (3-years OS: 55.1 % vs 82.6 %, *P* = 0.093).

### Inhibition of Notch signaling and proliferation in CC cells treated with GSI

**Fig. 2:**
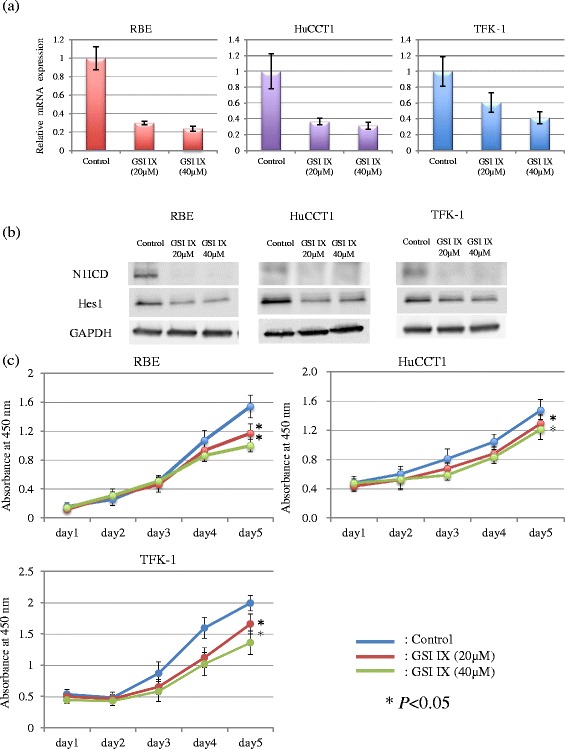
Alteration of Hes-1 expression and cell proliferation by GSI IX treatment in vitro. **a** qPCR. **b** Western blotting. Cleaved Notch1 (N1ICD) and Hes-1 expression was decreased in all cell lines treated with GSI IX, especially after exposure to 40 μM of GSI IX (**a**, **b**). **c** Proliferation Assay. GSI IX significantly reduced viable RBE, HuCCT1 and TFK-1 cells in a dose and time dependent manner (*P* < 0.05)

**Fig. 3:**
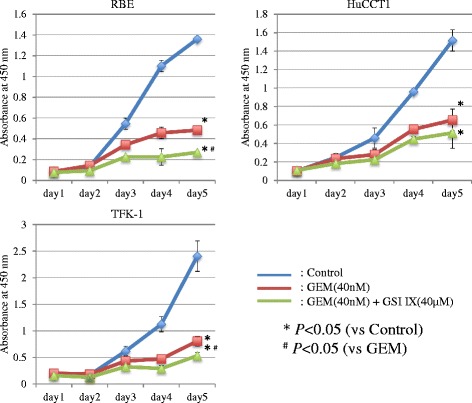
Alteration of cell proliferation by GSI IX treatment in the CC cell lines. The combination treatment of GSI IX and GEM significantly reduced viable RBE and TFK-1 cells compared with GEM monotherapy

To determine whether GSI could modulate Notch target genes, we assessed the alteration of Hes-1 expression in the CC cells lines by qPCR and Western Blotting. As illustrated in Fig. 2a, b, cleaved Notch1 (Notch1 intracellular domain: N1ICD) and Hes-1 expression was decreased in all cell lines treated with GSI IX, especially after exposure to 40 μM of GSI IX. Next, the effect of GSI IX on the proliferation of CC cell lines was determined by CCK-8 assay. GSI IX significantly reduced viable RBE, HuCCT1 and TFK-1 cells in a dose and time dependent manner (*P* < 0.05) (Fig. 2c). These results demonstrated that Notch signaling was related to the proliferation of CC cells. In the proliferation of CC cells, the combination therapy of GSI (40 μM) and GEM (40nM) significantly reduced viable RBE and TFK-1 cells compared with GEM monotherapy (Fig. 3).

### Alteration of subpopulation of CD24^+^CD44^+^ cells by GSI

**Fig. 4:**
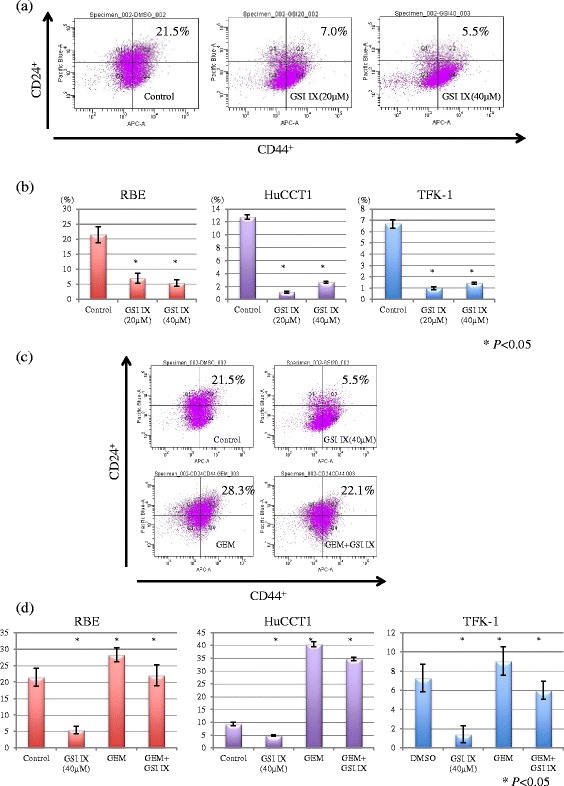
Alteration of subpopulation of CD24^+^CD44^+^ cells by GSI IX treatment in the CC cell lines. **a** Representative data in RBE cells treated with DMSO or GSI IX. **b** Percentage of CD44^+^CD24^+^ subpopulation in the CC cell lines exposed to GSI IX. The subpopulation of CD24^+^CD44^+^ cells after treatment with 20 and 40 μM of GSI IX were significantly decreased to 7.0 and 5.0 %, respectively, in RBE cell lines, compared to control (21.5 %) (*P* < 0.05). In the other CC cell lines, GSI treatment also decreased the subpopulation of CD24^+^CD44^+^ cells. **c** Representative data in RBE cells treated with DMSO, GSI IX, GEM or combination of GSI IX and GEM. **d** Percentage of CD44^+^CD24^+^ subpopulation in the CC cell lines treated with DMSO, GSI IX, GEM, or combination of GSI IX and GEM. The subpopulation of CD24^+^CD44^+^ cells increased to 28.3 % after monotherapy with GEM. The combination with GSI IX and GEM significantly diminished the subpopulation to 22.1 % (*P* < 0.05). The results of GEM monotherapy or combination of GSI IX and GEM were consistent in the other CC cell lines

We assessed the alteration in the subpopulation of CD24^+^CD44^+^ cells by treatment with GSI IX (Fig. 4a, b). Cells with CD24^+^CD44^+^ after treatment with DMSO were 21.5 %. The subpopulation of CD24^+^CD44^+^ cells after treatment with 20 and 40 μM of GSI IX were significantly decreased to 7.0 and 5.0 %, respectively, in RBE cell lines, compared to control (21.5 %) (*P* < 0.05). In the other CC cell lines, GSI treatment also decreased the subpopulation of CD24^+^CD44^+^ cells (Fig. 4b).

In contrast, the subpopulation of CD24^+^CD44^+^ cells increased to 28.3 % after monotherapy with GEM in RBE cell lines. The combination with GSI IX and GEM significantly diminished the subpopulation to 22.1 % (*P* < 0.05) (Fig. 4c). The results of GEM monotherapy or combination of GSI IX and GEM were consistent in the other CC cell lines (Fig. 4d).

### Anchorage-independent growth and mice tumorigenicity of GSI-pretreated cells

**Fig. 5:**
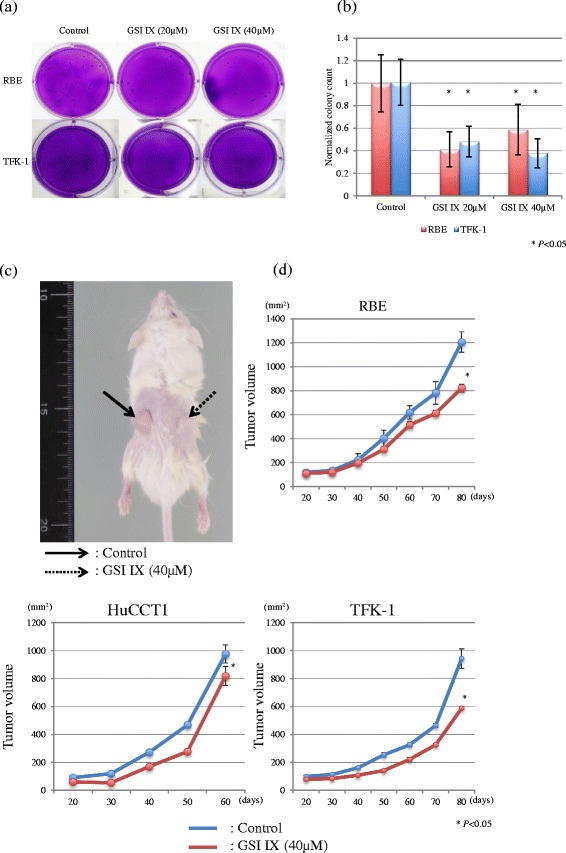
Anchorage-independent growth and mice tumorigenicity in the CC cell. lines pretreated with GSI IX ex vivo. **a** Colony formation in soft agar in RBE cells. **b** Normalized colony counts in RBE and TFK-1 cells pretreated with GSI IX. Pretreatment with both 20 and 40 μM of GSI IX significantly inhibited colony formations in the CC cell lines (*P* < 0.05). HuCCT1 cells did not form any colonies in soft agar after DMSO or GSI IX pretreatment (data not shown). **c** Representative photograph of engraftments of TFK-1 cells pretreated with GSI IX in NOD/SCID mice. Solid and dotted line arrow indicates GSI IX and DMSO, respectively. **d** Tumor growth curves of mice implantation in the CC cell lines with pretreatment of DMSO and GSI IX, respectively. Mice tumorigenicity in GSI IX pretreatment significantly delayed in all cell lines, compared to DMSO

To confirm the effectiveness of treatment with GSI on cells with CSC-like properties, we investigated the alteration of anchorage-independent growth and mice tumorigenicity after pretreatment with GSI IX (Fig. 5a–d). The ability to form clones in soft agar was inhibited more strongly by the pretreatment of GSI IX 20 μM and 40 μM, compared with DMSO-pretreated cells in RBE and TFK-1 (Fig. 5a, b). HuCCT1 cells did not form any colonies in soft agar after DMSO or GSI IX pretreatment (data not shown). As with the results of the anchorage-independent growth, the mice tumorigenicity of cells pretreated with GSI IX was significantly attenuated in all cell lines compared to DMSO (Fig. 5c, d).

## Discussion

Recently, several reports have discussed the participation of Notch signaling in CC [18–20]. However, the roles of Notch signaling in CC have not been fully understood. In the current study, IHC of resected EHCC specimens demonstrated aberrant expression of Notch1, 2 or 3 in approximately 40–60 % of the cohort and that of Hes-1 was found in approximately 80 %. This is the first report on aberrant Hes-1 expression in resected CC specimens. According to the confirmation of the Hes-1 expression, the activated Notch signaling in EHCC was endorsed. Moreover, the results of IHC implied that the expressions of Notch1 and 3 were associated with poorer histologic differentiation. Although there was no significant prognostic difference in the expression of each Notch receptor, the patients with the expression of at least any one of the Notch1, 2 and 3 tended to exhibit poorer survival, as well as those with the expression of Hes-1. Therefore, aberrant Notch signaling might be an indicator of poor survival. Yoon et al. reported the up-regulation of Notch1 and 3 in the progression of tumor stage in EHCC [17]. However, in our study, Notch1, 2, 3 and Hes-1 were not associated with the UICC stage. Thus, our results imply that Notch signaling participates in the initial step. Yoon et al. also described the high immunopositivity of Notch4 in EHCC [17], whereas our results showed very low positivity. Wu et al. described that positive immunostaining of Notch1 and 4 in IHCC were detected in 82.9 and 34.1 %, respectively, and were related to the tumor progression [16]. On the other hand, Notch1 and 2 were reported to play important roles in tumor proliferation and invasion in IHCC cell lines [18–20, 28, 29]. Accordingly, the types of Notch receptors involved in CC differ between the previous reports and the current study, the reason for which needs to be clarified in further studies.

GSI inhibits the γ-secretase-dependent cleavage of all Notch receptors as a pan-Notch inhibitor. GSI induces apoptosis through the regulation of nuclear factor-κB [32] and inhibits cancer cell growth and invasion. The effectiveness of GSI on cells with CSC-like properties has been reported in pancreatic cancer, breast cancer and brain tumor [21, 22, 33, 34]. Also, preclinical evidence in vivo has been demonstrated in some malignancies [21, 22]. A recent report clarified that CD24^+^CD44^+^ cells showed CSC-like properties in EHCC [35]. In this study, GSI exposure diminished the subpopulation of CD24^+^CD44^+^ in all CC cell lines and induced a significant reduction of anchorage-independent growth and delayed tumor engraftment in mice. The present study first elucidated the therapeutic effect of GSI on CC cells with CSC-like properties, similar to the effect found in other cancers.

The CSCs hypothesis is based on the idea that cancer tissue has a minute proportion of cells with stem cell-like properties, which possess a great ability for self-renewal and produce heterogeneous progeny. CSCs, which drive tumorigenesis and maintain tumor proliferation, are located at the top of a hierarchy of tumor cells [25–27]. Cells with CSC-like properties are activated after hypoxia exposure [36, 37], which is closely associated with multiple pathological microenviroments of CC, and considered to be resistant to conventional anticancer therapies. To fail to eradicate cells with CSC-like properties ultimately results in relapse even when conventional therapy shows a dramatic effect. Hence, in addition to conventional therapies, the successful eradication of cells with CSC-like properties is needed. The systematic chemotherapy of GEM plus CDDP is now proposed as the most promising therapy for unresectable CC [9, 10]. This study showed resistance to GEM in CC cells with CSC-like properties. In a previous report, pancreatic cancer cells with CSC-like properties had resistance to GEM in a patient-derived xenograft model [38]. Although GEM is suggested to be generally ineffective against cells with CSC-like properties, the combination therapy of GEM plus GSI decreased the subpopulation of CD24^+^CD44^+^ cells compared with GEM alone. Combination therapy of GEM plus GSI was reported to show a synergy effect in pancreatic cancer xenograft [21, 22]. Moreover, a recent report described that treatment of lung cancer with erlotinib, which is an epidermal growth factor receptor (EGFR) tyrosine kinase inhibitors, resulted in dramatic cell death and paradoxical enrichment of cells with CSC-like properties through EGFR-dependent Notch signal activation [39]. Notch signaling may cause therapy-induced resistance because of its cross-regulation with other oncogenic pathways. Therefore, additional GSI treatment has potential as a new therapeutic strategy in CC.

## Conclusions

Aberrant Notch signaling is involved in EHCC. In CC, inhibition of Notch signaling could be a novel therapeutic strategy for targeting cells with CSC-like properties.

## Abbreviations

BSA: Bovine serum albumin
CC: Cholangiocarcinoma
CDDP: Cisplatin
CSCs: Cancer stem cells
DMSO: Dimethyl sulfoxide
ECOG: Eastern Cooperative Oncology Group
EDTA: Ethylenedinitrilotetraacetic acid
EGFR: Epidermal growth factor receptor
EHCC: Extrahepatic CC
FBS: Fetal bovine serum
GEM: Gemcitabine
GSI: γ-secretase inhibitor
Hes-1: Hairy and enhancer of split 1
IHC: Immunohistochemistry
IHCC: Intrahepatic CC
NICD: Notch intracellular domain
NOD/SCID: Nonobese diabetic/severe combined immunodeficiency
OS: Overall survival
PBS: Phosphate buffered saline
PMSF: Phenylmethanesulfonyl Fluioride
pNET: Pancreatic neuroendocrine tumors
PVDF: Polyvinylidenefluoride
qPCR: Quantitative real-time reverse transcription polymerase chain reaction
SAB: Streptavidin-biotin
SDS: Sodium dodecyl sulfate
UICC: Union for the International Cancer Control
